# Trapped as a Group, Escape as a Team: Applying Gamification to Incorporate Team-building Skills Through an ‘Escape Room’ Experience

**DOI:** 10.7759/cureus.2256

**Published:** 2018-03-02

**Authors:** Xiao Chi Zhang, Hyunjoo Lee, Carlos Rodriguez, Joshua Rudner, Teresa M Chan, Dimitrios Papanagnou

**Affiliations:** 1 Department of Emergency Medicine, Thomas Jefferson University; 2 Faculty of Health Sciences, Department of Medicine, Division of Emergency Medicine, McMaster University

**Keywords:** medical education, teamwork, communication, escape room, graduate medical education, faculty development, simulation

## Abstract

Teamwork, a skill critical for quality patient care, is recognized as a core competency by the Accreditation Council for Graduate Medical Education (ACGME). To date, there is no consensus on how to effectively teach these skills in a forum that engages learners, immerses members in life-like activities, and builds both trust and rapport. Recreational ‘Escape Rooms’ have gained popularity in creating a life-like environment that rewards players for working together, solving puzzles, and completing successions of mind-bending tasks in order to effectively ‘escape the room’ in the time allotted. In this regard, escape rooms share many parallels with the multitasking and teamwork that is essential for a successful emergency department (ED) shift.

A pilot group of nine emergency medicine (EM) residents and one senior EM faculty member underwent a commercial escape room as part of a team-building exercise in January 2018. The escape room required participants to practice teamwork, communication, task delegation, and critical thinking to tackle waves of increasingly complex puzzles, ranging from hidden objects, physical object assembly (i.e., jigsaw puzzles), and symbol matching. Activities required members to recognize and utilize the collective experiences, skills, knowledge base, and physical abilities of the group. After the game, players underwent a structured ‘game-master’ debriefing facilitated by an employee of the commercial escape room; this was followed by a post-event survey facilitated by a faculty member, which focused on participants’ feelings, experiences, and problem-solving techniques.

Escape rooms afford learners the opportunity to engage in an activity that rewards teamwork and effective leadership through experiences that directly link to specific ACGME milestones and educational learning theories. EM participants were engaged in the activity and felt that the escape room reproduced an environment analogous to the ED. The debriefing that followed the activity provided a satisfactory conclusion to the experience; but learners preferred a more organized debriefing format that provided them with constructive and specific feedback on their performance.

## Introduction

Teamwork is arguably one of the most difficult skills to teach learners in the health professions, especially in chaotic and unpredictable environments, such as the emergency department (ED). Team members must overcome varied levels of training and expertise, conflicting personalities, and diverse skill sets to effectively function as a unit. Studies continue to demonstrate that effective teamwork and collaboration, particularly in high-stakes, high-acuity environments, have the ability to decrease mortality and cost [1, 2]. Trust has also been cited as a critical teamwork element in the military literature, in which a team with an established level of trust can effectively communicate and function with surgical precision [3].

Training opportunities in teamwork must be safe, low-stakes, high-impact, and dynamically-engaging if they are to nurture the development of collaborative behaviors. Although teamwork remains a core competency by the Accreditation Council for Graduate Medical Education (ACGME), there is no consensus as to how to effectively teach this skill. Effective team training demands receptive and engaged learners, immersive learning activities and experiences, and participant trust and rapport.

Committed to creating a fun and interactive team training activity, the investigators turned to the growingly popular ‘Escape Room’ as a medium to immerse learners in an environment similar to the ED. Escape Rooms are live-action, team-based games where players must work together, solve clues, and complete a series of mind-bending, fitness-challenging tasks in order to ‘escape the room’ in under 60 minutes [Nicholson S. (2015). Peeking behind the locked door: a survey of escape room facilities. White paper available at http://scottnicholson.com/pubs/erfacwhite.pdf]. Players (or learners) must learn how to quickly acclimate themselves in a foreign environment with other players (possibly strangers) of various skill sets, establish trust, and communicate specific goals and tasks in order to assess the environment, analyze clues, solve complicated problems, reassess the environment, and set-up additional goals and dispositions. To a certain extent, this experience is analogous to caring for real patients in an ED setting. While several effective team-building exercises are described in the healthcare literature [4-7], there is currently no mention of the use of commercial escape rooms as opportunities for team training for ED providers.

In this technical report, the investigators describe the use of an escape room as a pilot team-building exercise for providers working in the ED of Thomas Jefferson University Hospital in Philadelphia, Pennsylvania. The investigators contracted with a local escape room agency to allow 10 participants from the ED to participate in a one-hour escape room session, followed by a post-event debriefing.

## Technical report

Study settings

The commercial escape room agency was selected based on convenience of location, given its proximity to the hospital (less than 1 mile). The average rate of successful ‘escapes’ at this location is measured by the agency to be 20%. Four unique escape room formats are available at this particular location, each with a unique theme and unique set of puzzles and challenges. Each escape room also contains multiple sub-rooms to sequentially escape from as the activity unfolds.

At the end of the game, players underwent a structured, escape-room debriefing, in which the escape room ‘game-master’ described the puzzles and respective solutions; this allowed players to recount their experiences and problem-solving techniques. The observer (XCZ) did not provide any post-game feedback, nor did he participate in the debriefing.

Study participant selection

Participants were selected on a first-come, first-serve basis, after a flyer that advertised the activity was electronically mailed to ED residents and faculty. The activity was advertised three weeks in advance. The investigation was reviewed and approved by the Institutional Review Board of Thomas Jefferson University.

Materials required

Participants were not required to bring any additional tools or materials to the escape room.

Group structure

Ten participants were assigned to a single group without any predetermined roles or task designations. Their only assigned group goal was to effectively escape the room within 60 minutes. One of the study investigators (XCZ) also entered the escape room to observe team behaviors and record team interactions; he did not participate in the activity, and participants were asked to ignore his presence.

Detailed activity description

To escape the room, participants must practice teamwork, communication, task-delegation, critical thinking, and divergent thinking to tackle a series of increasingly complex puzzles, ranging from hidden objects, physical object assembly (i.e., jigsaw puzzles), counting, and/or symbol matching. Throughout the activity, the game master watches the participants’ progress through several cameras that are physically mounted throughout the room. Players may receive sporadic visual hints, displayed on a television monitor in the room, which are provided by the game master if participants either fail to progress in solving clues or if participants wish to directly receive a clue.

Specific puzzle information and details surrounding the escape room were unknown to the authors and the participants prior to reserving the escape room, as this information is considered proprietary knowledge to the escape room agency.

Activity evaluation

The investigators used a validated, post-study survey evaluation adapted from a post-focus group interview guide in order to obtain written feedback about the escape room activity [8]. The referenced survey was developed and successfully used at McMaster University for postgraduate EM resident physicians to assess a modular assessment program. The escape room questionnaire consists of 18 questions, which are included in Appendix A. Responses from the questionnaire were evaluated via an open-axial qualitative assessment by XCZ, HL, and CR.

Results

Ten total participants (eight second-year EM residents, one third-year EM resident, and one EM faculty member) participated in this activity. There were approximately 10 to 12 unique puzzles with topics ranging from, but not limited to, recognizing hidden objects, symbol substitutions, mathematical challenges, searching for objects in images, rearranging objects based on visual aids, light manipulation, assembly of a physical object, riddles, and pattern identification. The team received only three hints throughout the activity (average per agency, 10-15) and escaped the room in 46 minutes (average success rate per agency, 20%).

A post-activity survey analysis (n=10) revealed that eight out of 10 participants had previously participated in escape room activities. Everyone, however, had previously participated in a team-building activity, with ‘sports’ being the most common activity (nine out of 10 participants). All participants reported similarities between the escape room and the ED, with common themes focusing on chaos, communication, strategic thinking, differential diagnoses, teamwork, uncertainty, task delegation, and time constraints. Participants also contrasted the escape room to the ED, highlighting that the escape room felt more linear, clue-based, and low stakes (Table 1).

**Table 1:** Selected Participant Survey Responses Following the Escape Room ED = Emergency Department

Survey Questions	Common Themes
Q4 - Please recall the interaction you had with your colleagues in the game. Were they similar to tasks you perform in the ED?	Task Switching; Task Delegation; Data Compilation; Plan Development; Task Specification; Disposition Planning
Q5 - Please recall the interaction you had with your colleagues in the game. Were they similar to the interactions you have had with your applications in the ED?	Polite; Taking Turns; Independent Data Collection; Collective Brainstorming and Data Sharing
Q6 - Please recall the debriefing conducted by the game master at the conclusion of the game. Was this similar to the debriefing at the end of your shift?	Different than Healthcare Debriefing; Inadequate Debriefing; Different Debriefing Style
Q10 - What are some immediate lessons you learned by working with your peers in the Escape Room?	Contribution of Unique Skills; Appreciation of Skill Development Outside the Workplace; Organic Practice of Leadership Skills
Q17 - If you were to redesign this team-building activity, what would you want to include to make it a more effective activity that focuses on team work and communication?	Fewer Sequential Puzzles; More Concurrent Puzzles; Diversifying Participants; More Comprehensive Debriefing with Constructive Feedback

Nine out of 10 participants endorsed similarities in the social and interpersonal interactions that take place in both the game and the ED; these include co-managing stressful situations, settling differences in opinion, and providing periodic status updates. While all of the participants appreciated the post-escape room debriefing, half of the participants would have preferred a more structured debriefing, similar to a debriefing following a simulation, which typically includes formative feedback based on observed behaviors. Nine participants reported that the escape room experience motivated them to learn more about teamwork, specifically how to overcome barriers to teamwork, how to manage conflicting personalities (i.e. introverts vs. extroverts), how to multitask, and how to resolve personal differences. All participants rated the escape room as an effective, fun, teambuilding activity, and even recommended including additional puzzles and additional personnel of varied professions (i.e., nurses, technicians) and varied levels of training for heightened realism.

## Discussion

The authors propose that commercial escape rooms can be utilized as effective team building activities by immersing participants in interactive, unpredictable, and chaotic environments that can encourage team members to exercise their unique skill sets to seek out and solve different challenges. Escape rooms share several similarities to the clinical environment, which make them successful learning adjuncts for members of clinical teams.

Open-axial qualitative assessment of the survey responses revealed common themes in clinical (i.e., ED) and escape-room interactions, such as task switching, role delegation, and working together towards a common goal. Additional common themes included a preference for fewer sequential puzzles (versus simultaneous challenges that occur in parallel) to more closely align with the multiple demands that are expected when working in an ED. Participants would have also preferred a more organized and constructive debriefing.

The educational philosophy of this activity is rooted in behaviorism, social learning, and cognitivist and constructivist learning theories. In behaviorism, ‘positive’ behavior is reinforced by providing progression through the escape room process, whereas ‘negative’ behavior either does not help the players (i.e. learners) escape the room or even negatively impacts their ability to escape (Table 2). There is a large aspect of social learning in this activity, as learners imitate or perpetuate behaviors of successful puzzle solving. Learners can also assimilate their previous knowledge and skills (i.e. pattern recognition, mathematical fluency, trivia knowledge) to help them process environmental clues. The latter is akin to the cognitivist approach to learning.

Aspects of constructivism are also embedded in this activity: learners construct their own knowledge based on real-time experiences of advancing through several challenges in the escape room. In accordance with the ACGME EM Milestones, escape rooms also afford the learners the opportunity to practice several milestones: Patient Care (PC8 – Task Switching) and Interpersonal Communication Skills (ICS1 – Interpersonal and Communication Skills and ICS2 – Leading Patient-Centered Care Teams) (Table 2). In order to escape the room, each player must demonstrate the ability to switch tasks between assimilating clues, solving puzzles, and reassessing the environment under high-stress situations using flexible communication strategies. Participants must coordinate their efforts to solve complicated puzzles and recommend changes in their team to establish optimal efficiency (i.e. redistributing the team members from focusing on one puzzle to searching for additional clues). These challenges could not be more similar to a busy Monday shift in the ED.

**Table 2:** Accreditation Council for Graduate Medical Education (ACGME) Milestones Specific to Emergency Medicine (EM) with Corresponding Learning Theories Found in an Escape Room Activity ACGME = Accreditation Council for Graduate Medical Education PC = Patient Care ICS = Interpersonal and Communication Skills Source: https://www.acgme.org/Portals/0/PDFs/Milestones/EmergencyMedicineMilestones.pdf [9]

ACGME Milestone	Escape Room Activity	Relevant Educational Learning Theories
PC8: Task Switching	Level 1: Solves a single puzzle amidst a chaotic environment; Level 2: Pays attention to discovered clues while completing a task; Level 3: Actively communicates with team members to share new hints or solutions; Level 4: Actively reassesses environment to assign new tasks after a stage is completed	Constructivist: Players use previous puzzle-solving knowledge to help process clues. ​​​​​Cognitivist: Players acquire new knowledge based on real-time puzzle solving
ICS1: Interpersonal and Communication Skills	Level 1: Establishes rapport with fellow players; Level 2: Negotiates and manages simple conflicts during the game; Level 3: Effectively communicates with struggling players to minimize stress when engaged in a complex puzzle; Level 4: Uses flexible communication strategies to assign appropriate tasks to assist with puzzle-solving	Social Learning: Players imitate puzzle-solving behaviors with success. Behaviorism: Positive behavior is reinforced by progression in the escape room
ICS2: Leading Patient-Centered Care Teams	Level 1: Participate as an individual player; Level 2: Communicates pertinent clues and/or solutions to the team leader; Level 3: Demonstrates clear communication with team members; Level 4: Recommends changes in team behaviors to solve complicated puzzles (i.e. assigns a new player to a problem or considers an alternate approach)	Social Learning: Players imitate puzzle-solving behaviors with success. Behaviorism: Positive behavior is reinforced by progression in the escape room

There are several limitations worth noting. While this activity was originally designed to be an inclusive team-building event that was open to all eligible ED staff, the study investigators were unable to accrue a diverse group of participants within the enrollment period due to scheduling conflicts. Furthermore, commercial escape rooms can be expensive, making it costly to conduct this activity with a large group of students, residents, and/or faculty. It is possible, however, to divide larger groups into more manageable sizes of six to 10 learners. The presence of an observer in the escape room may have led to a Hawthorne effect, which can be addressed in future iterations using the surveillance recorders available in the escape room. Finally, the debriefing was limited to the post-activity discussion facilitated by the agency's game master; future team-building exercises may benefit from a standard debriefing, focusing on observed behaviors and results to provide learners with constructive feedback. An economical alternative to commercial escape room is to construct an emergency department-themed escape room with EM-specific puzzles and challenges set in a private residence with each room representing different locations in the ED. This allows the creators and participants to become more engaged in puzzle solving and enables more constructive feedback.

## Conclusions

The escape room team-building activity was well received by our EM residents and faculty. It allowed learners to immerse themselves in an engaging, fun, non-threatening, non-clinical, low-stakes activity that rewarded teamwork and effective leadership. Next steps will include expanding interprofessional inclusion (i.e., nurses and technicians), as well as designing escape rooms with more complex puzzles that require parallel processing to closely approximate the chaotic nature of the ED. The authors found that while the game debriefing provided insight into actions taken during the game, participants would have preferred a more constructive feedback session to help them analyze aspects of communication and problem-solving that can be applied in subsequent scenarios, including the clinical environment.

## Appendices

Appendix A – Post escape room survey questionnaire

1. Have you ever used an escape room?

1a. If yes to question #1

1b. What were your initial reactions to it?

1c. I’d like you to compare your expectations to when you were first introduced to it and now, after having had some time working with the system. Was it any different from what you expected?

2. Have you ever participated in team-building activities (i.e. team steps, sports activities)?

2a. If yes, what were the activities?

3. I invite you to think about your clinical settings, how do those environments compare to the escape room?

3a. Can you identify any differences or similarities?

4. Please recall the tasks you performed in the escape room, were these similar to the tasks you perform in the emergency department (ED)?

5. Please recall the interaction you had with your colleagues in the game, were they similar to the interactions you have with your applications in the ED?

6. Please recall the debriefing conducted by the game master at the conclusion of the game, were they similar to the debriefing at the end of your shift?

6a. Why or why not?

7. How do you prepare for your clinical shifts?

8. How do you prepare for the clinical team interactions you have on shifts?

9. How do you prepare for the debriefing that takes place at the end of the shift?

10. What are some immediate lessons you learned by working with your peers in the escape room?

10a. Do you find this experience useful? Why or why not?

11. Thinking back, which experience from the escape room will motivate you to learn more about working in a team?

12. Thinking back, which experience from the escape room will dissuade you from working in a team?

13. What is your perception of teamwork and communication in emergency medicine?

14. How do you determine the value of effective teamwork and communication?

15. How do you determine the value of debriefing at the end of a clinical shift?

16. Was the escape room activity an effective team building activity?

16a. What made it effective?

16b. How will you use this information?

17. If you were to redesign this team building activity, what do you want to include to make it a more effective activity that focuses on team work and communication?

17a. Prompt: What will you want to take out or change?

17b. Prompt: Why would you implement that change?

18. Do you have any questions for me? Are there topics that we’ve yet to cover? Is there anything you want to discuss?

## References

[1] Clemmer TP, Spuhler VJ, Oniki TA, Horn SD (1999). Results of a collaborative quality improvement program on outcomes and costs in a tertiary critical care unit. Crit Care Med.

[2] Baggs JG, Ryan SA, Phelps CE, Richeson JF, Johnson JE (1992). The association between interdisciplinary collaboration and patient outcomes in a medical intensive care unit. Heart Lung.

[3] Adams B, Sartori J (2006). Trust in Teams: A review of the Literature. Report to the Defence and Civil Institute of Environmental Medicine. http://www.dtic.mil/dtic/tr/fulltext/u2/a630700.pdf.

[4] Yi YJ (2016). Effects of team-building on communication and teamwork among nursing students. Int Nurs Rev.

[5] Kumar S, Deshmukh V, Adhish VS (2014). Building and leading teams. Indian J Community Med.

[6] Ndulue U, Peréa FC, Kayou B, Martinez LS (2012). Team-building activities as strategies for improving community-university partnerships: lessons learned from Nuestro Futuro Saludable. Prog Community Health Partnersh.

[7] Stoller JK, Rose M, Lee R, Dolgan C, Hoogwerf BJ (2004). Teambuilding and leadership training in an internal medicine residency training program: experience with a one-day retreat. J Gen Intern Med.

[8] Li SA, Sherbino J, Chan TM (2017). McMaster Modular Assessment Program (McMAP) through the years: residents' experience with an evolving feedback culture over a 3-year period. Acad Emerg Med Educ Train.

[9] (2017). The Emergency Medicine Milestone Project. https://www.acgme.org/Portals/0/PDFs/Milestones/EmergencyMedicineMilestones.pdf.

